# Coronavirus Disease 2019 (COVID-19): Prevention and Control in Gynecological Outpatient Clinic

**DOI:** 10.3389/fpubh.2020.618494

**Published:** 2021-01-08

**Authors:** Dongmei Yao, Kun Yan, Jie Duan, Xian Zhang, Limin Zhou

**Affiliations:** ^1^Department of Gynecology, Maternal and Child Health Hospital of Hubei Province, Tongji Medical College, Huazhong University of Science and Technology, Wuhan, China; ^2^State Key Laboratory of Virology, Modern Virology Research Center, College of Life Sciences, Wuhan University, Wuhan, China

**Keywords:** infection control, transmission, COVID-19, gynecological public health, gynecological practice management

## Abstract

**Objective:** The pandemic of coronavirus disease 2019 (COVID-19) has become a major public health challenge around the world, and outbreaks of the SARS-CoV-2 have constituted a public health emergency of international concern. Infection control measures are necessary to prevent further spread of the virus and to help control the epidemic situation. Due to the characteristics of gynecological settings, the risk of cross infection between patients and gynecologic practitioners can be high, strict and effective infection control protocols are urgently needed. This article, based on our experience and relevant guidelines and research, introduces prevention and control measures for use in gynecological outpatient clinics and provides recommended management for gynecologists in (potentially) affected areas.

## Introduction

On 31 December 2019, the cases of pneumonia of unknown etiology were firstly reported in Wuhan City. The number of people infected with the novel coronavirus subsequently named as SARS-CoV-2 dramatically increased worldwide, and the virus causing coronavirus disease (COVID-19) was characterized as a pandemic (1, 2). SARS-CoV-2 can be transmitted among humans through droplets and contacts (2–4). There is also the possibility of aerosol transmission and fecal-oral transmission (5). Patients with mild symptoms exhibit fever, dry cough and fatigue. Severe patients may have dyspnea, respiratory failure and severe infection. Our hospital is located in Wuhan, the most serious epidemic area in China. Total confirmed cases of COVID-19 were over 50,000 in Wuhan, it accounted for more than half of the total number of confirmed cases of China. However, there are only eight confirmed cases in Wuhan on 25 November 2020 (Data sources: WHO, CDC and local media reports). With improvement of the domestic epidemic situation, outpatient services are gradually recovering, but the global epidemic status is not encouraging. However, during the epidemic, there were still patients with gynecological problems requiring outpatient treatment. Gynecological patients required gynecological examinations, and some of them needed further procedures. Although no evidence of the SARS-CoV-2 can be found in the vagina, SARS-CoV-2 viral nucleic acids can be detected in the blood (6, 7). Moreover, healthcare workers are at risk of contracting respiratory virus infections when delivering routine care for patients infected with the viruses, and they are at risk of disseminating virus because they touch virus-contaminated fomites (8–10). Gynecological healthcare workers have close contact with patients, and the risks should not be underestimated. To prevent contagion during the epidemic, our gynecology clinic has formulated prevention and control measures and workflows during the epidemic. From January 23 to May 23, 2020, 27,212 patients were treated, 3,357 patients and their families were screened, and 14 cases were abnormal (positive for nucleic acid or SARS-CoV-2 antibody IgM). There was no infection among the healthcare workers of the gynecology clinic. The results are summarized as follows.

## Prevention and Control Measures in Outpatients

### Patient Management

The outpatient service adopted an online appointment system. Patients with appointment information and their family members (only 1 person) were able to see a doctor. Patients and their families were asked to wear masks, and correct mask wearing was emphasized (11). The entrance of the hospital was equipped with an infrared temperature detector. The staff at the entrance used a hand-held temperature gun to measure the temperature of the people who entered the hospital. If the body temperature was higher than normal, they were admitted to the fever clinic. Individuals with normal body temperature could enter. Before entering the gynecological area, the triage nurse checked the health codes of the patients and their families. Based on the mobile big data provided by government, key persons, subjected to epidemic prevention and control, are indicated with red code. Persons at risk of potential epidemic are with yellow code. Other persons are indicated with green code. The patients with a green code could see a doctor, and the patients with a red code were advised to go to designated hospitals. The temperature was measured again. Then, the triage nurse marked the normal temperature and green code symbol on the treatment sheet. The patients filled out the outpatient screening form “outpatient 14 questions” (Table 1) at the triage table. Items 1–4 were epidemiological history screening, items 5–7 were clinical symptom screening, and items 8–14 were related to high risk factor screening. The patients were asked if they had high risk factors, fever, cough and other acute respiratory illness symptoms. The patients were required to fill out the form and sign the notification before entering the diagnosis room. In the diagnosis room, the doctor reconfirmed the temperature and questionnaire and then began the outpatient clinic work. Gynecological patients are usually accompanied by family members. Except for patients in emergency departments, only patients were allowed to enter the waiting area, and accompanying individuals were required to wait outside the waiting area. The safety distance between personnel was required to be more than 1.5 meters, and we marked 1.5 m intervals on the floor (12). There are subsequent phone contacts with patients and their family after leaving the hospital at 7th and 14th day, to confirm whether they have been diagnosed as positive for COVID-19. If a case of COVID-19 is confirmed, the clinical staff who has contacted the COVID-19 patient, as well as the patients who have contacted the staff during that period, will be tracked according to the records in the hospital's computer system. These persons at potential epidemic risk will subject to viral nucleic acid test and put in quarantine for 14 days (Table 2).

**Table 1:** Epidemiological investigation of COVID-19.

1	Have you lived in a community with reported cases within 14 days?
2	Have you had contact with a covid-19 infected person (nucleic acid test positive) within 14 days?
3	Have you been exposed to someone with fever or respiratory symptoms within 14 days?
4	Do you have 2 or more confirmed covid-19 cases or asymptomatic infections around you?
5	Have you had a fever in 14 days?
6	Do you have cough, expectoration, suffocation, sore throat, chest pain and other respiratory symptoms within 14 days?
7	Do you have fatigue, myalgia or diarrhea within 14 days?
8	Have you ever taken a plane, train, coach or been to a designated hospital within 14 days?
9	Have you ever attended a party with more than 2 people or been to a public place with more than 2 people in 14 days?
10	Are you a front-line anti epidemic personnel (front-line medical staff in designated or shelter hospitals, staff involved in epidemic disposal and exposed to confirmed or suspected cases)?
11	If they are frontline anti epidemic personnel, have they met the following three conditions after finishing their work (isolation for 14 days, no abnormality in CT Reexamination, and negative nucleic acid test)?
12	Have you ever been a confirmed or suspected patient?
13	If you have ever been a confirmed or suspected patient, did you meet the following four conditions (cured and isolated at the isolation point for 14 days, and then isolated at home for 14 days after returning home, no abnormality in CT Reexamination and negative nucleic acid test)?
14	Is there any other special situation or additional explanation needed? If yes, please inform the medical staff.
	**Signature: Tel: Address: **
**Notes:**	For your and everyone's public medical safety, please answer the above questions truthfully! Otherwise, if there are serious consequences, you will be investigated for legal responsibility according to the law of the people's Republic of China on the prevention and control of infectious diseases and other relevant laws. If necessary, it will be handed over to the judicial organ for filing and handling the crime of endangering public security by dangerous means. I have answered the above questions truthfully, without any concealment. If there is any concealment, I will bear the corresponding legal responsibility!

**Table 2:**
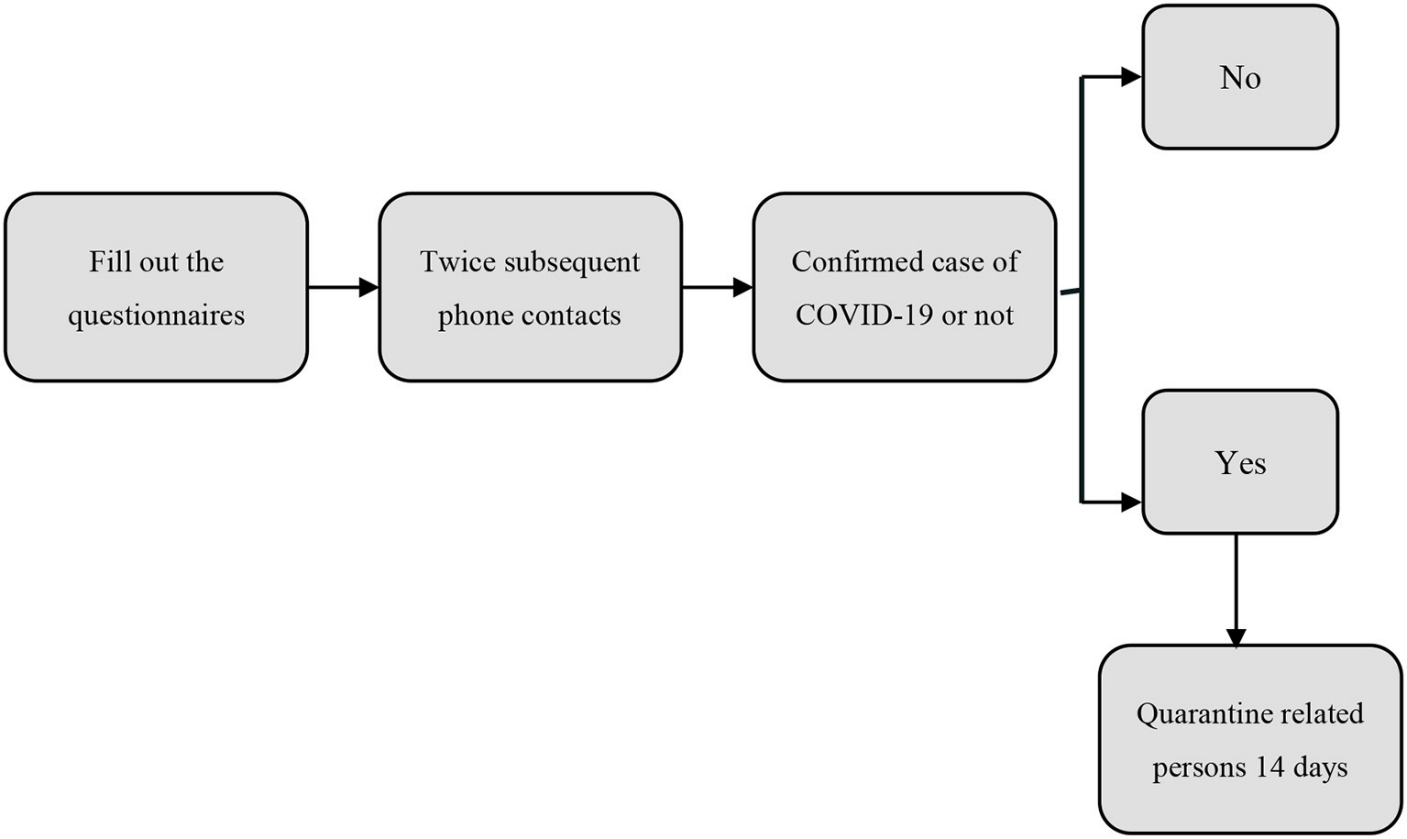
Track condition of patients work flow.

### Management of Gynecological Healthcare Workers

The temperatures of gynecological healthcare workers were measured before work and any abnormal results were reported immediately. It's necessary to ensure complete hand hygiene, physical distancing and use other personal protective equipment (PPE), including masks, gloves, gowns, cap, shoe cover, and goggles or face shield, to prevent dissemination of virus. Triage nurses and doctors in the gynecology outpatient department adopted measures of no less than two-level protection. In gynecological procedure areas, such as colposcopy rooms, gynecological treatment rooms, hysteroscopy rooms, family planning procedure rooms, etc., the staff wore three-level protection, including isolation clothes, masks, gloves and shoe covers, goggles and face screens. Hand hygiene and proper mask wearing were emphasized to reduce the risk of SARS-CoV-2 pneumonia among healthcare workers. During lunch and rest time, healthcare workers were required to maintain an effective distance of more than 1.5 meters. The indoor ventilation was maintained in good condition, and staff were scheduled to eat in batches to avoid large gatherings. In addition, triage nurses and doctors accepted viral nucleic acid and antibody tests irregularly. Once a local positive case is confirmed, all clinical staff have to be screened.

### Site and Equipment Management

**Figure 1:**
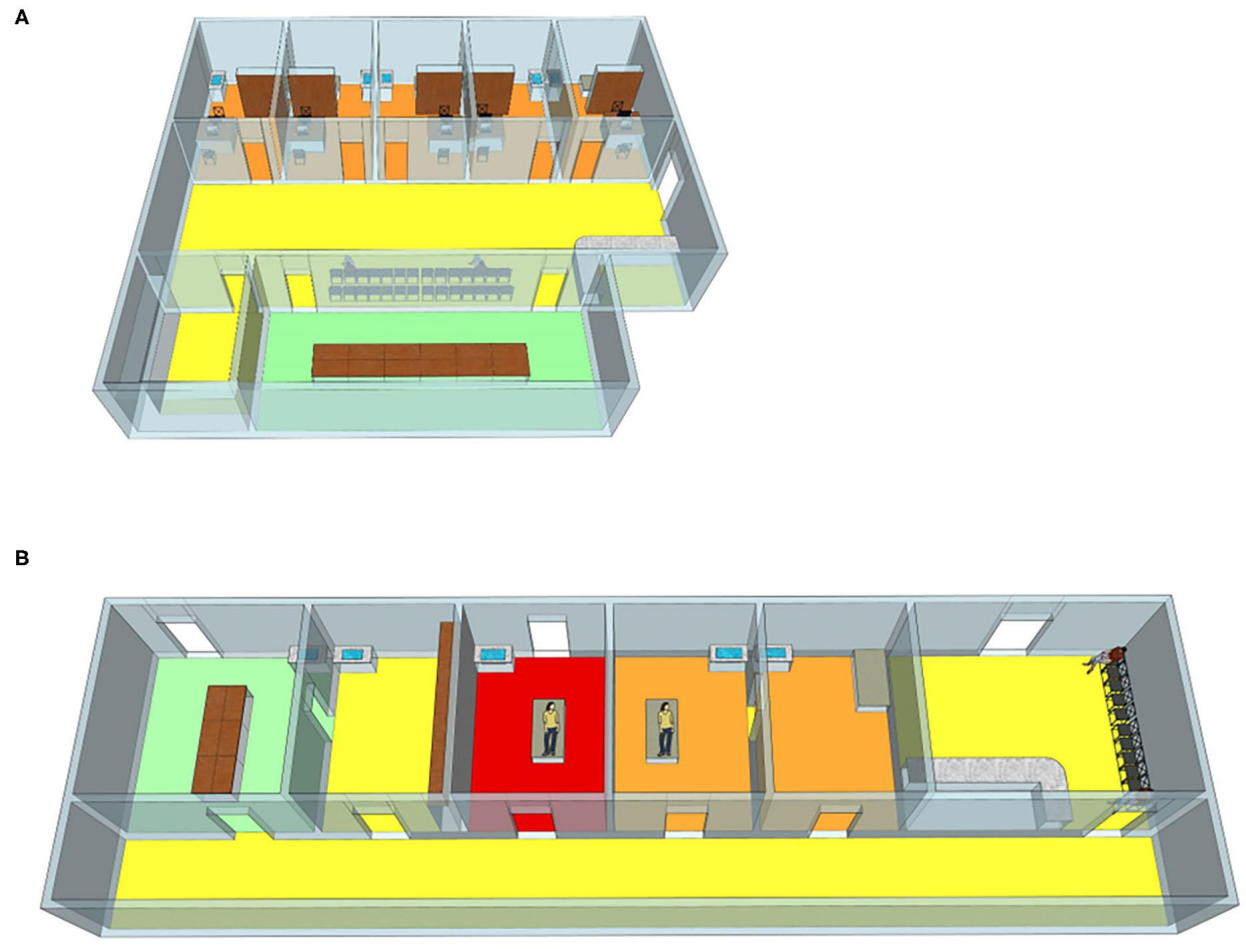
The outpatient district **(A)** and operation room **(B)**. Yellow: triage and waiting area. Orange: Gynecology clinic and operation room. Red: isolation operation room. Green: resting area for staff only. As shown in the diagram, our triage staff in the yellow area wear disposable surgical mask, cap, and work clothes. In the orange area, gynecologic staff is provided with PPE (personal protective equipment), including disposable N95 masks, gloves, gowns, cap, shoe cover, and goggles or face shield. The area is disinfected once every half day. All the patients were treated in this area. The isolation clinic in the red area is designed for patients who are suspected with COVID-19 or who are recovering from COVID-19. Separate entrances for patients. gynecologic staff should wear protective clothing besides the aforementioned PPE. In addition, the entire isolation area is disinfected immediately after the treatment is over and the patient has left. Staff can have a rest in the room (green area). They are recommended to enter the room by turn and to keep wearing medical masks unless they are eating or drinking.

To decrease the risk of nosocomial infection, patients could be treated in an isolated and well-ventilated room (Figure 1). In addition, central air conditioning was stopped in the waiting area, diagnosis room and outpatient operating rooms, and ventilation was strengthened (13–15). To allow for natural ventilation, windows and doors may remain open for 30 min, at least twice a day. Besides, it is also able to control and guarantee airflow by installing a fresh air system. According to our country's technical standards, the minimum ventilation rate of the biosafety laboratory is 12 times/hour and the minimum ventilation rate of the negative pressure isolation ward is 8 times/hour. Supplement general ventilation with air disinfector and germicidal ultraviolet lights. If a patient suspected of infection was found in the clinic, for example, if a positive test for SARS-Cov-2, viral pneumonia antibody IgM, or typical CT changes were detected, the clinic room was changed immediately and disinfected with ozone. After lunch break and after work each day, an ozone disinfector was used to disinfect the diagnosis room and the operating rooms of the outpatient department. In the outpatient waiting area, a 3% hydrogen peroxide spray was used to disinfect the air. Object surfaces were wiped with 1,000 mg/L chlorine containing disinfectant or disinfected by ultraviolet radiation. It was reported that SARS-CoV-2 has been detected in anal swabs (16, 17), although no SARS-CoV-2 has been found in the vagina. However, in gynecological examination, the female reproductive system contacts the pad sheet directly and may contaminate the examination table. Therefore, attention is needed during gynecological examination in particular to avoid cross infection. The patients were examined with disposable pads, one each, and sodium hypochlorite (effective chlorine content: 1,000 mg/L) were used to wipe and disinfect the examination bed and operating bed directly in contact with patients. A rapid hand spray (alcohol-based hand rub) was placed in the guide table and diagnostic room for disinfection at any time.

## Characteristics of Gynecological Clinics During the Epidemic Period

### Outpatients and Disease Classification From January 23, 2020 to May 23, 2020

**Figure 2:**
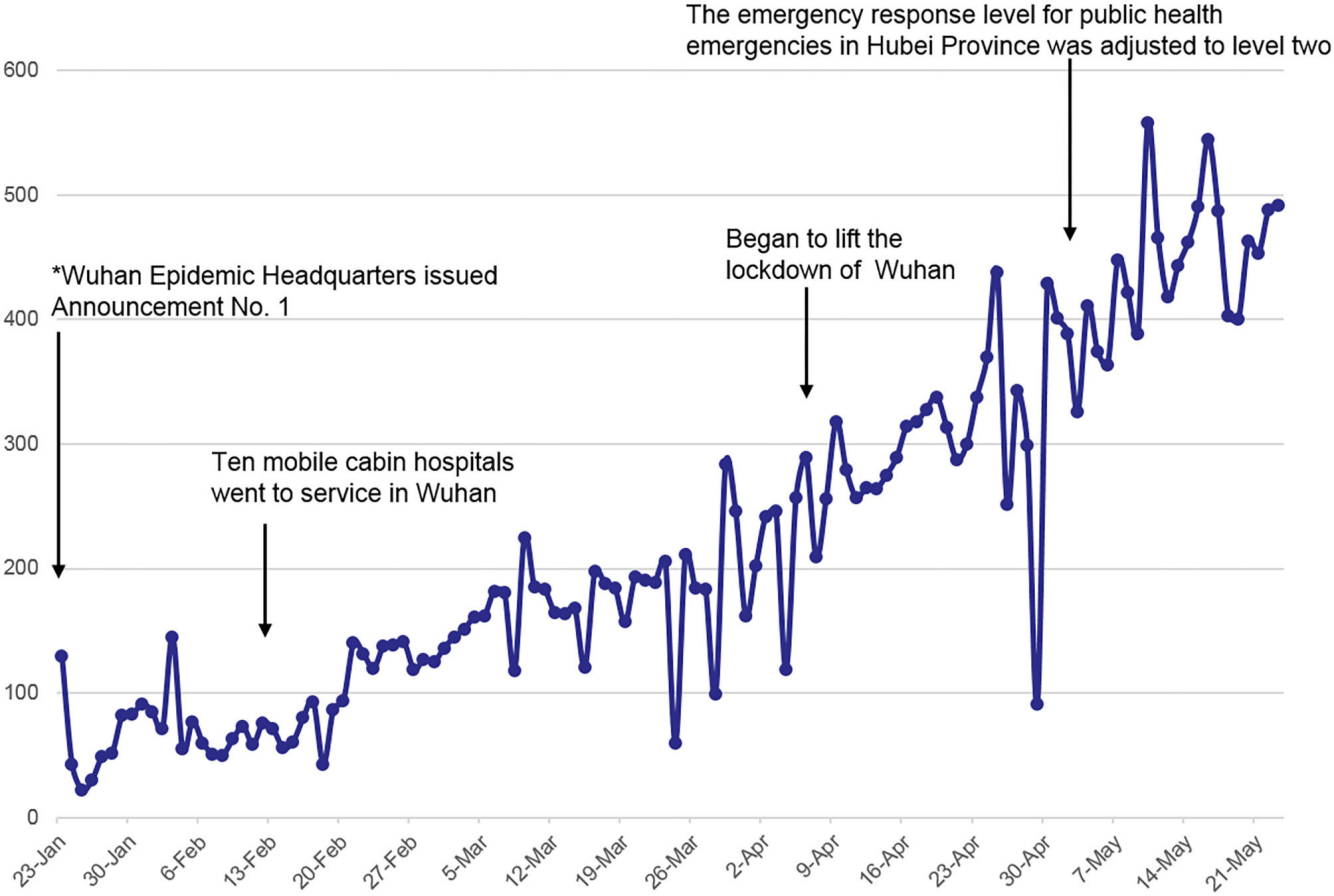
Number of outpatients treated at the Maternal and Child Health Hospital of Hubei province from January 23, 2020, to May 25, 2020. *From 10:00 a.m. on January 23, 2020, the city's urban bus, subway, ferry and long-distance passenger transportation would be suspended, the departure channels of airport and railway stations be temporarily closed. As the condition of the hospital gradually returned to normal, patients traveled smoothly, and the outpatient volume come to recovered.

A total of 27,212 patients were treated in the gynecology department (Figure 2). Before Wuhan was unsealed on April 8, 2020, most of the patients were diagnosed with pregnancy-related conditions, including threatened abortion, missed abortion, early pregnancy (termination of pregnancy), and ectopic pregnancy, which accounted for more than 95% of the total number of outpatients. Gynecological emergencies include abnormal uterine bleeding, rupture of the corpus luteum, rupture of ectopic pregnancy, inevitable abortion and massive hemorrhage.

### Screening Work Flow

Patients who needed to be admitted to the hospital or to undergo outpatient surgery [family planning surgery, outpatient cervical endoscopy, laser surgery, loop electrosurgical excision procedure (LEEP) surgery, tubal hydrotubation] were required to undergo the following examinations (Table 3): CT lung screening, routine blood tests and CRP, SARS-CoV-2 nucleic acid and SARS-CoV-2 pneumonia antibody. If typical ground glass changes were observed on CT, the patient must go to the fever clinic; if the nucleic acid or SARS-CoV-2 antibody IgM test is positive, the patient was isolated immediately and the result reported to the medical department. If only the antibody IgG test was positive, the patient was advised to take another nucleic acid test. If the patient is in an emergency situation and needed to be hospitalized immediately, the patient was admitted to the buffer room of the ward for treatment. If necessary, only one fixed person was permitted to accompany the patient, and this person was required to undergo CT, routine blood tests, CRP, SARS-CoV-2 nucleic acid and SARS-CoV-2 antibody tests. If the above results were normal, they were permitted to enter the ward to accompany the patient. We screened 3,357 cases, 14 of which had abnormal results (including positive tests for SARS-CoV-2 nucleic acid or SARS-CoV-2 antibody IgM). They were transferred to designated hospitals for further examination.

**Table 3:**
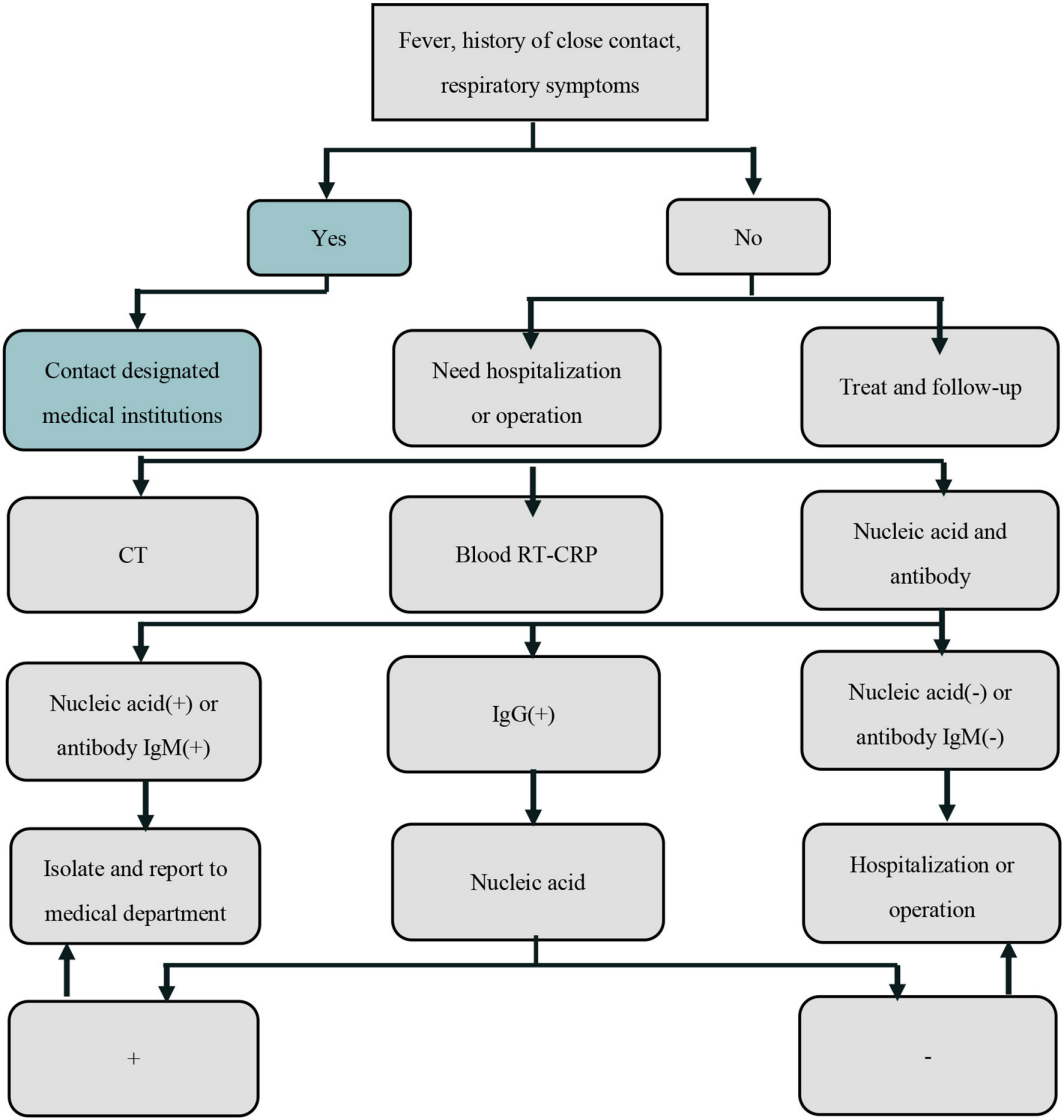
Screening work flow.

### Characteristics of Outpatient Operation

All specimens that may be considered potential risks for biological contamination, such as leucorrhea specimen test tubes, cervical cytology specimen bottles, and pathological examination specimens, were placed in specimen bags for inspection. The outpatient family planning operating room was divided to provide a special area for patients in need of emergency treatment with no time for screening. In strict compliance with the above procedures for prevention and control and screening, none of the healthcare workers in the gynecological outpatient department was infected.

## Discussion

### Importance of Prevention and Control Measures in Gynecological Outpatient Clinics

After the outpatient temperature screening, medical history inquiry, health code inquiry and temperature screening again, the patients with symptoms were basically eliminated and could not enter the gynecological outpatient department. However, asymptomatic infection could not be completely ruled out. Asymptomatic patients are infectious, and we should try to identify such patients. The hospital environment was relatively enclosed; after entering the inpatient department, there was a high chance of spreading the epidemic. SARS-CoV-2 is highly contagious. The main routes of transmission are respiratory droplets and close contact transmission. There may also be air transmission and fecal-oral transmission. Although there is no evidence showing the existence of SARS-CoV-2 in the vagina, the patients and doctors shared the same space with limited air flow, and there was a possibility of infection. SARS-CoV-2 nucleic acids can be detected in blood; thus during outpatient procedures, such as family planning surgery, hysteroscopy, and tubal fluid infusion, the healthcare workers may be contaminated by blood. Therefore, outpatient doctors have a certain risk of infection, and outpatient control is very important.

### Gynecological Outpatient “Infection Control” Measures During the Epidemic Period

The goals were to establish treatment and screening procedures for patients during the epidemic, reduce cross infection among patients, family members and healthcare workers, and ensure patients obtain safe and appropriate medical services. Patients were encouraged to seek medical treatment online and open telemedicine channels. Attention was given to the training of healthcare workers, combined with relevant guidelines and consensus recommendations, so that healthcare workers could master the protection procedures and constantly improve the treatment process. Outpatient patients used an appointment system, and patients with appointments made online were permitted to enter the outpatient hall. One patient was permitted per consultation room to reduce the number of people gathered together. In the process of treatment, it was important to pay attention to protective measures for the healthcare workers, disinfect the examination bed immediately, and put biologically contaminated specimens into specimen bags for inspection. Patients who needed to be admitted to the hospital and those who were to undergo outpatient surgery were screened according to the outpatient process.

### Changes in Gynecological Outpatient Services During the Epidemic Period

During the epidemic period, due to the closed management of each community, it was not easy for patients to go out. Therefore, all the patients who came to the hospital were in emergency situations. The main types of problems in the outpatient department were threatened abortion and early pregnancy. There was abnormal uterine bleeding, inevitable abortion, ectopic pregnancy, rupture of the corpus luteum and other emergencies that needed to be treated immediately. The patient's illness, as well as worry about the epidemic situation, made the patient's mood more anxious, so the outpatient healthcare workers should be more patient than usual. Outpatient work became more cumbersome than before. Triage nurses needed to measure body temperature, collect outpatient questionnaires, etc., and the time to process a single patient became significantly longer than before, especially for patients with surgical needs or the need for hospitalization. Patients and their families sometimes needed multiple follow-up visits according to the outpatient screening process, and the waiting time for results was increased. Patients could turn to the emergency department during the waiting period. Therefore, the process should be fully explained before the patient visits so that the patient can understand the process and provide informed consent to avoid disputes caused by repeated examinations.

During the outbreak of SARS-CoV-2, disease prevention and control measures should be standardized as part of the treatment process, which is conducive to the safe continuation of gynecological outpatient work. We have described the working setting of the gynecology outpatient clinic. We hope our experience can help other gynecological colleagues overcome the epidemic as soon as possible.

## Data Availability Statement

The raw data supporting the conclusions of this article will be made available by the authors, without undue reservation.

## Ethics Statement

The studies involving human participants were reviewed and approved by the Medical ethics committee of the Maternal and Child Health Hospital of Hubei Province. Written informed consent for participation was not required for this study in accordance with the national legislation and the institutional requirements.

## Author Contributions

LZ and DY conceptualized the study design. DY, JD, and XZ designed the work flow and collected data. DY, KY, and JD plotted the figures and analyzed the data. DY wrote the initial drafts of the manuscript. KY and XZ revised the manuscript. All authors contributed to the article and approved the submitted version.

## Conflict of Interest

The authors declare that the research was conducted in the absence of any commercial or financial relationships that could be construed as a potential conflict of interest.
